# Prevalence of markers of HIV infection among febrile adults and children in Bo, Sierra Leone, 2012–2013

**DOI:** 10.1186/s13104-017-2912-2

**Published:** 2017-11-06

**Authors:** Rashid Ansumana, Donald F. Dariano, Kathryn H. Jacobsen, Tomasz A. Leski, Chris R. Taitt, Joseph M. Lamin, Joseph Lahai, Umaru Bangura, Alfred S. Bockarie, Chadwick Yasuda, Moses J. Bockarie, David A. Stenger

**Affiliations:** 1 Mercy Hospital Research Laboratory, Kulanda Town, Bo, Sierra Leone; 2 Department of Community Health and Clinical Studies, Njala University, Bo, Sierra Leone; 3 Center for Biomolecular Science & Engineering, U.S. Naval Research Laboratory, Washington, DC USA; 4 Department of Global & Community Health, George Mason University, Fairfax, VA USA; 5 Liverpool School of Tropical Medicine, 5 Pembroke Place, Liverpool, L35QA United Kingdom

**Keywords:** HIV, Surveillance, Immunological tests, Sierra Leone, West Africa

## Abstract

**Objective:**

The goal of this study was to examine the prevalence of HIV among febrile patients seeking care in Mercy Hospital, Bo, Sierra Leone, in 2012–2013.

**Results:**

A total of 1207 febrile persons were tested for HIV with Determine™ and SD Bioline rapid diagnostic tests kits that detect the presence of HIV antibodies and HIV p24 antigens. The overall prevalence of HIV among the tested patients was 8.9%, which is considerably higher than the < 2% prevalence of HIV reported previously in the general population. While these results are not sufficient to prove a causal relationship, the obtained data imply that HIV positive individuals may be more likely to suffer from febrile infectious diseases than individuals without HIV infection. Increasing the availability and use of HIV testing services will allow antiretroviral therapy to be accessed in a timely manner and improve health status among people living with HIV.

## Introduction

HIV/AIDS was first detected in Sierra Leone in 1987 among commercial sex workers in the Bo and Kenema districts [1]. In 1993, the Sierra Leone National AIDS Control Programme reported an 8.4% national seroprevalence rate [2]. That study was conducted during a period of civil war in Sierra Leone (1991–2002), when rape and multiple perpetrator rape were commonplace [3]. Surveys undertaken as the civil conflict was ending showed decreasing rates of HIV seroprevalence [4]. However, the inability of researchers to access participants from all demographics during this time of conflict led to concern that the sampled population did not accurately represent the national population as a whole.

In April 2002, the first national survey of HIV prevalence in Sierra Leone was conducted by the Sierra Leone Ministry of Health in collaboration with the U.S. Centers for Disease Control and Prevention (CDC). Based on specimens collected from 2412 persons aged 12 to 49 years from 724 households, the weighted HIV seroprevalence rate was 0.9% [5]. Since that time, government-sponsored surveys reported national HIV prevalence in the range of 1–2% [6, 7], with the most recent survey reporting a 1.5% HIV positive rate for the general population [8]. However, while HIV rates of the overall population and of several key high-risk populations have been well documented [9], little is known about the burden of HIV among individuals with febrile illnesses.

A higher rate of acute infectious diseases among individuals with HIV than those without HIV infection would suggest that HIV is not being adequately managed by antiretroviral medication. The primary goal of this analysis was to investigate the prevalence of HIV among febrile patients in Bo, Sierra Leone, using rapid HIV diagnostics. Fever remains a common symptom of primary HIV and the opportunistic infections that affect people living with HIV [10]. A previous study within the study location reported a 25.9% (95% CI 24.8–27.1%) prevalence of self-reported febrile illnesses including HIV within a 6 month period [11].

## Main text

### Methods

The study was a part of a single-center, longitudinal, prospective study conducted at the Mercy Hospital Research Laboratory in Bo, Sierra Leone, during the 1-year period from July 1, 2012, through June 30, 2013. The goal of the larger study was to determine the etiology of febrile disease within Mercy Hospital’s catchment area. Mercy Hospital offers inpatient treatment, but the vast majority of its patients consult with clinicians at the hospital’s outpatient clinic. Local residents aged 6 years and older presenting to staff at Mercy Hospital with febrile symptoms or reporting a fever that began within the previous 7 days were eligible to participate. Febrile individuals were defined as persons with an oral temperature of 37.5 °C or greater or an axillary body temperature of 38 °C or greater [12]. Those excluded from the study were local residents who were unwilling or unable to provide blood samples and children who for whom parental consent (and, for older children, assent) were not obtained.

Whole blood samples collected from 1207 consented febrile individuals participating in the prospective cohort study were tested for HIV with the Determine™ HIV-1/2 lateral flow immunoassay (LFIA) test (471 samples; Alere Medical Co., Matsudo-shi, Chiba, Japan) and/or the SD Bioline 4th Generation HIV Ag/Ab Combo LFIA (736 samples; Standard Diagnostics Inc. Kyonggi-do, Korea). Both test kits meet the World Health Organization’s recommended standards for in vitro diagnostic products [13]. The Determine™ test kit detects the presence of HIV antibodies with a 100% sensitivity and a 98.9% specificity [14]. The SD Bioline test kit detects both HIV antibodies and HIV p24 antigens, and it is reported to have a 99.8% sensitivity and a 100% specificity [13, 15]. HIV antibodies are typically indicative of chronic HIV infection, while the presence of p24 is a marker for the acute HIV infection that occurs in the weeks immediately following the initial exposure to the virus.

Blood samples were collected by trained and qualified research staff. The assays were performed according to the manufacturer instructions. A Deki Reader (Fio Corporation, Toronto, Canada) was used to capture the LFIA images and upload them to a cloud-based database hosted by Fio Corporation. The final determination of the outcome of the assays was based on the visual inspection of both the original tests by on-site researchers and uploaded images accessed remotely by team members located in the USA. Any positive test was repeated and also redone with the other brand of rapid test.

Epi Info software (CDC, Atlanta, USA) was used to estimate the sample size and to conduct statistical tests. Chi squared tests with a two-sided significant level of α = 0.05 were used to evaluate statistical comparisons between various subpopulations by age group and sex.

### Results

A total of 1207 febrile individuals (456 males and 751 females) were tested for HIV using one or both of the rapid diagnostic devices. The overall prevalence of the presence of HIV antibodies was 8.3% (100/1207) (Table 1). Although the seroprevalence of HIV antibodies ranged from 4.1 to 12.3% within different age groups, these differences were not statistically significant (p = 0.483). Male and female participants were equally likely to be HIV-seropositive (p = 0.379). HIV antigen p24 consistent with early HIV infection was detected in 8 of 736 tested individuals (1.1%; Table 2), with no significant age- or sex-specific differences (p > 0.335). As expected, all antigen-positive patients were negative for the presence of HIV antibodies.

**Table 1:** Prevalence of HIV antibodies within febrile individuals, 2012–2013 (SD bioline and determine HIV Ag/Ab LFIAs)

Age group	Total			Male			Female		
	Number tested	Number positive	% HIV+	Number tested	Number positive	% HIV+	Number tested	Number positive	% HIV+
6–14	88	4	4.5	39	2	5.1	49	2	4.1
15–29	460	34	7.4	140	10	7.1	320	24	7.5
30–44	265	27	10.2	111	9	8.1	154	18	11.7
45–59	181	12	6.6	78	7	9.0	103	5	4.9
60+	213	23	10.8	88	12	13.6	125	11	8.8
Total	1207	100	8.3	456	40	8.8	751	60	8.0

**Table 2:** Prevalence of p24 antigens among febrile individuals positive for HIV, 2012–2013 (SD Bioline HIV Ag/Ab LFIA)

Age group	Total			Male			Female		
	Number tested	Number positive	% HIV+	Number tested	Number positive	% HIV+	Number tested	Number positive	% HIV+
6–14	48	1	1.4	21	1	4.8	27	0	0.0
15–29	265	4	1.0	87	3	3.4	178	1	1.6
30–44	153	1	0.5	67	0	0.0	86	1	1.2
45–59	108	0	0.0	43	0	0.0	65	0	0.0
60+	163	2	1.0	68	1	1.5	95	1	1.1
Total	736	8	0.8	286	5	1.7	451	3	0.4

## Discussion

The overall prevalence of HIV infection among febrile individuals was 8.9% after including all participants who were positive for either antibody or antigen in the numerator of the calculation. This rate is almost six times greater than the 2012–2013 national HIV survey that found an HIV prevalence rate of 1.5% for the general population and 1.4% for the population in Bo district [16]. Since the LFIAs used for our study generally require the presence of higher concentrations of HIV for a positive test than is required for the ELISA-based methods used in the national survey [17], the actual difference in the HIV rates in the current study of febrile patients and the national survey of the general population may be even bigger.

The large disparity between our results and those of the 2012–2013 national survey suggests a significant difference between the populations sampled for each of the studies. Variations in HIV prevalence based on age, socioeconomic group, level of education, and other demographic characteristics may be partly responsible for the higher rate of HIV-positives in the current study [8]. However, the more likely cause of the large discrepancy in HIV rates is the intentional use of only febrile patients in this study. Several other studies in low-income countries have reported higher rates of HIV infection among febrile individuals than non-febrile individuals [18–20]. Fever is commonly associated with acute HIV infection (AHI), a febrile illness occurring shortly after HIV infection and characterized by the presence of HIV nucleic acids and/or p24 antigen, but not antibodies. However, in our study only small fraction of all subjects were positive for p24 (8/108) but not antibodies. These individuals may all have had acute HIV infections, but we did not confirm this with the CD4^+^ counts that are typically used to evaluate disease progression to AIDS.

While a causal link cannot be made, the more plausible explanation of the large number of febrile patients seropositive for HIV antibodies suggests that HIV-infected individuals may have a higher incidence of non-AHI related febrile illnesses than people without HIV infection. These results are likely a consequence of a current gap in HIV testing and treatment in Sierra Leone, despite three decades of government efforts to prevent HIV infection and spread [21]. In Bo, where this study was carried out, about two-thirds of young adults have never sought testing for HIV, including 56% of women and 75% of men [22]. The low testing rate means that many adults with HIV remain unaware of their HIV status and unaware of their need to seek HIV treatment. This data, combined with the fact that only 29% of individuals who are aware of their HIV-positive status in Sierra Leone receives antiretroviral therapy (ART) [23], points to a potentially large pool of people lacking proper management of their HIV infection.

Perhaps the most striking observation from this study is the high rate of HIV infection in children ages 6–14 years, most of whom are presumed to have contracted HIV from their mothers [24]. Although we note that these results must be interpreted cautiously based on the limited number of children (n = 88) who participated in the study, the 4.1% rate observed among febrile children in this study is more than eight times greater than the 0.5% prevalence reported for this age group in the general population in 2015 [23]. This high rate in children was unexpected, since about 80% of HIV-positive pregnant women in Sierra Leone receive ART [23]. ART reduces the rate of mother-to-child transmission to less than 2% if received during pregnancy and delivery [24]. This finding suggest that HIV infection may be associated with increased incidence of acute febrile infections in pediatric populations as well as adult populations.

Overall this study shows that almost one in eleven febrile patients reporting to Mercy Hospital is HIV seropositive. This suggests that HIV-related infections may account for a high proportion of use of the health system nationally. The contribution of HIV to the overall burden of disease may be underestimated after factoring in the excess risk of comorbid infectious diseases among people with HIV infections. Additional funding for HIV management through ARTs may reduce the health system costs of treating acute and chronic secondary infections.

These findings also point toward the value of clinical referrals for HIV voluntary counselling and testing (VCT). In Sierra Leone and other places where a large proportion of people with HIV do not know their status and are not receiving ART, it might be appropriate for health authorities to recommend that all febrile adults be referred for VCT. Since these individuals are already seeking care for their illnesses, they might be more likely to accept the referral and follow through on testing, receipt of the results, and follow-up care if the HIV test is positive. This kind of testing might also facilitate detection of acute HIV infection and enhance opportunities to control HIV spread.

## Limitations

The major limitation of this study is that the participants consisted entirely of a convenience sample of individuals with acute febrile illnesses who were not randomly sampled from the population, and we did not include a comparison group to verify the < 2% HIV prevalence rate in the general (afebrile) population in the Mercy Hospital catchment area. We also did not measure CD4 counts or attempt to classify the stage of HIV infection among participants with positive HIV tests. Additionally, we did not have a large enough sample size to have the statistical power necessary for robust comparisons of HIV prevalence rates between various age-sex subpopulations.

Despite the limitations, the results may provide helpful information about the burden of acute febrile illnesses among people with HIV infection who live in Bo and, more generally, in Sierra Leone. Future studies that assess the prevalence of HIV among randomly-selected subjects from general population are necessary to accurately assess the extent of the HIV epidemic in Sierra Leone, but it is likely that people with HIV infection account for a disproportionately high number of patients seeking diagnosis and treatment for acute undifferentiated febrile illnesses.

## Abbreviations

Ab: antibody
Ag: antigen
AHI: acute HIV infection
AIDS: acquired immune deficiency syndrome
ART: antiretroviral therapy
CDC: US center for disease control
CI: confidence interval
HIV: human immuno-deficiency virus
LFIA: lateral flow immunoassay
p: probability
VCT: voluntary counselling and testing
